# Effect of ferric-carboxy maltose on oxygen kinetics and functional status in heart failure patients with iron deficiency

**DOI:** 10.2144/fsoa-2019-0156

**Published:** 2020-03-31

**Authors:** Sandip Dhoot, Sanjay Mittal, Simar Pal Singh, Vishal Patel, Ravi R Kasliwal, Varshil Mehta

**Affiliations:** 1 Department of Cardiology, Dedanta, The Medicity Hospital, Gurgaon, India; 2 Chelsea & Westminster Hospital NHS Foundation Trust, West Middlesex University Hospital Site, Isleworth, UK

**Keywords:** ejection fraction, ferric-carboxy maltose, ferritin, heart failure, iron deficiency, MLHFQ score, New York Heart Association (NYHA) functional class, peak VO_2_, quality of life (QoL), six-minute walk distance

## Abstract

There is a very high prevalence of iron deficiency anemia in patients with systolic heart failure. The present study is a prospective, parallel, 1:1 randomized controlled trial of intravenous ferric-carboxy maltose compared with standard of care in patients with heart failure. A total of 70 patients who presented to us with symptomatic chronic heart failure were included and randomly assigned to either groups (35 per group). Post 12 weeks, there were improvements noticed in peak VO_2_, New York Heart Association functional classification, 6-min walk test distance covered and reduction in Minnesota Living with Heart Failure Questionnaire score in the ferric-carboxy maltose as compared with standard of care group. However, no improvement in ejection fraction was noticed.

## Lay abstract

Iron deficiency is commonly seen in patients with heart failure. This study was performed to observe the effect of intravenous iron therapy (ferric-carboxy maltose) in this population. The outcome showed significant benefit in symptoms and improvement in quality of life. These results are in concordance with other similar trials. Therefore, simple intravenous iron replacement along with other heart failure measures can make life easier for patients with heart failure.

Anemia is present in approximately 40% of heart failure patients and is defined as <12 g/dl in women and <13 g/dl in men. The major cause of anemia worldwide is iron deficiency, which is observed in almost 60% of anemic patients [1]. Furthermore, approximately 50% of heart failure patients suffer from iron deficiency (irrespective of their anemic status). This proportion further increases to 81.6% in the Indian population [2].

Due to such a high prevalence rate, recent literature and international guidelines have begun suggesting that iron deficiency should be addressed as a comorbid condition among systolic heart failure patients [1].

The treatment of iron deficiency anemia was initially focused on oral hematopoietic agents. However, heart failure patients have been shown to exhibit abnormal intestinal morphology, which impairs oral iron uptake from the gut and inflammation induced hepcidin impairs iron delivery to bone marrow, which is why the anemia of chronic inflammatory disease does not respond to oral iron Fe (II) preparations [1]. In the absence of any iron treatment, 50% of patients with heart failure have been estimated to have insufficient iron availability for hematopoiesis [3].

In contrast to oral iron therapy, intravenous (IV) iron preparations have been reported to result in both clinical and statistical improvement in anemia, iron deficiency, renal function, improved left ventricular ejection fraction, left ventricular end systolic dimension, left ventricular end diastolic dimension, left ventricle mass index, reduced C reactive protein and NT-Pro BNP. They have also been shown to improve New York Heart Association (NYHA) functional class, the 6-min walk distance (6MWT) and quality of life [1].

Currently used IV preparations, such as ferric saccharate, ferric gluconate or ferric-carboxy maltose (FCM) are well tolerated [3]. They have been reported to effectively treat iron deficiency in heart failure (HF), but very little information is available on the efficacy in improving oxygen kinetics.

Although in the FERRIC HF trial, IV sucrose used over 16 weeks significantly improved the exercise tolerance (peak VO_2_) in heart failure patients with and without anemia (n = 35), the impact of IV FCM to improve oxygen kinetics and symptomatic status in the Indian population is still unknown.

Therefore, we designed a randomized control study (FCM-HF-IN) in an Indian population to test the hypothesis that iron repletion with intravenous FCM alone will increase peak VO_2_, which is a gold standard measure of exercise capacity in heart failure patients. Other outcomes measured were changes in levels of ferritin, hemoglobin (Hb), left ventricular ejection fraction, 6MWT, NYHA Class and the Minnesota Living with Heart Failure Questionnaire (MLHFQ) score.

## Materials & methods

The current study is a prospective, parallel, 1:1 randomized controlled trial of IV iron as FCM compared with standard of care (without iron). The patients included were symptomatic with chronic heart failure (NYHA Functional Class II/III) of age ≥18–65 years with iron deficiency, seen at Heart Failure Clinic Medanta Hospital, Gurgaon over a 2-year period (from June 2016 to June 2018). The study was approved by the institutional ethics committee of the Medanta, the Medicity Hospital.

The sample size was calculated according to data from the FERRIC-HF trial [4]. For randomization, a sequence of 70 random numbers (35 per group) was generated using computer software. After recruitment and signed consent, the allocation of sample to two groups was based on the sequence of random number kept in opaque envelopes.

Those with severe anemia (Hb<8 g/dl, requiring blood transfusion within 30 days), chronic liver disease, vitamin B12 deficiency (<200 pg/dl), serum folate deficiency (7 nmol/l) and/or any other significant cardiac disorder were excluded from the study. All data were recorded in preset proforma. A clinical examination followed by investigations including Hb concentration, reticulocyte count, serum iron, total iron binding capacity (TIBC), serum ferritin, transferrin, 12 lead-ECG, cardiopulmonary bicycle ergometer test, 6-min walk test, MLHFQ questionnaire and NYHA class were performed at baseline week 0 and at 12th week.

2D Doppler echocardiography was performed using standardized equipment (vivid ultrasound systems, General Electric, WI, USA) with adherence to a uniform image acquisition protocol. For cardiopulmonary bicycle ergometer test, all underwent maximal-effort cycle exercise testing using an incremental protocol on a cycle ergometer (Ganshorn Medizin Electronics, Niederlauer, Germany). For 6-min walk tests, participants were made to walk in a 54-m corridor complying with ATS Guidelines [5]. Total distance walked, time to breathlessness using stop watch timer, dyspnoea and fatigue levels was noted on Borg scale [6] and monitoring was performed with pulse oximeter.

After tests, IV FCM solution (Ferinject^®^, Vifor Pharma, Glattbrugg, Switzerland) was administered to eligible subjects in 0.9% normal saline bolus over 1 h (it was hospital policy to give intravenous iron over 1 h because patients are usually symptomatic and slow rate of infusion possibly reduces the risk of severe drug-related reactions and heart failure decompensation) via drug infusion pump while the control group received standard of care. Adverse events were managed and recorded throughout the study and till the last follow-up visit. All serious adverse events (decompensated heart failure, anaphylactoid reaction, death) were reported to an independent safety officer and the appropriate research governance committee within 24 h of occurrence.

Statistical analysis included profiling of patients for both the groups on different demographic and clinical parameters. Independent Student’s t-test was used for comparison of individual quantitative parameters between groups and paired t-test for within the group. P-value <0.05 was considered statistically significant. SPSS software, version 24.0 was used for statistical analysis.

## Results

A total of 70 patients were enrolled in this study and they were randomly assigned to two groups. Group 1 included those who received injectable FCM while group 2 included those who received standard of care (SOC). Table 1 shows the comparison of different parameters in both groups at baseline and at 12 weeks. The mean age in FCM group (group 1) was 51.0 ± 11.6 years, while in SOC group (group 2), it was 54.8 ± 9.0 years. (p = 0.150, not significant). The majority in both the groups were males, being 57.1% in group 1 and 60% in group 2. A total of 31.4% had previous history of coronary artery disease in group 1 while the percentage for this in group 2 was almost 26%. Serum ferritin (μg/l) at baseline in group 1 and 2 was 40.1 ± 27.2 and 45.5 ± 35.1, respectively. At 12 weeks, it rose to 162.7 ± 57.9 in FCM arm whereas it increased to a value of only 79.9 ± 49.8 in those receiving standard care (p < 0.0001). In contrast, serum hemoglobin did not show significant difference in values at baseline as well as at 12 weeks (p 0.249 and 0.236, respectively).

**Table 1.** Comparison between ferric-carboxy maltose and standard of care group in management of iron deficiency anemia in heart failure patients.

Parameters	Ferric-carboxy maltose group	Standard of care group	T value	p-value
Age (mean ± SD)	51.0 ± 11.6	54.8 ± 9.0	-3.714	0.150
Male (percentage)	57.1	60	–	0.808
Weight in kilograms (mean ± SD)	66.9 ± 10.3	67.5 ± 11.7	-0.224	0.823
Coronary artery disease (percentage)	31.4	68.6	–	0.597
Serum ferritin in μg/l at baseline (mean ± SD)	40.1 ± 27.2	45.5 ± 35.1	-0.720	0.474
Serum ferritin in μg/l at 12 weeks (mean ± SD)	162.7 ± 57.9	79.9 ± 49.8	6.419	<0.0001 (significant)
Serum hemoglobin in mg/dl at baseline (mean ± SD)	11 ± 1.4	11.3 ± 0.9	-1.164	0.249
Serum hemoglobin in mg/dl at 12 weeks (mean ± SD)	12.5 ± 1.2	12.2 ± 1.3	1.195	0.236
Ejection fraction at baseline (mean ± SD)	24.9 ± 5	25.8 ± 5.5	-0.750	0.456
Ejection fraction at 12 weeks (mean ± SD)	26.6 ± 4.9	27.1 ± 5.4	-0.392	0.696
Distance covered in meters in 6-min walk test at baseline (mean ± SD)	431 ± 64.79	418 ± 46.9	0.902	0.370
Distance covered in meters in 6-min walk test at 12 weeks (mean ± SD)	502.1 ± 70	455.5 ± 52.3	3.158	0.002 (significant)
MLHFQ score at baseline (mean ± SD)	46.9 ± 15.7	47.9 ± 15.1	-0.257	0.798
MLHFQ score at 12 weeks (mean ± SD)	34.7 ± 13	41.9 ± 13.2	-2.299	0.025 (significant)
NYHA at baseline	Class II – 26 (74.3%)	Class II – 30 (85.7%)	–	0.232
	Class III – 9 (25.7%)	Class III – 5 (14.3%)		
NYHA at 12 weeks	Class I – 26 (74.3%)	Class I – 17 (48.6%)	–	0.027 (significant)
	Class II – 9 (25.7%)	Class II – 18 (51.4)		
Peak VO_2_ in ml/kg/min at baseline (mean ± SD)	12.1 ± 3.1	12.1 ± 3	-0.057	0.955
Peak VO_2_ in ml/kg/min at 12 weeks (mean ± SD)	14.9 ± 3.4	12.9 ± 3.2	2.632	0.010 (significant)
Absolute VO_2_ in ml/min at baseline (mean ± SD)	801 ± 221.8	821 ± 267.9	-0.346	0.730
Absolute VO_2_ in ml/min at 12 weeks (mean ± SD)	993.9 ± 249.8	875.9 ± 301.1	1.784	0.079^†^

^†^p-value <0.05 was considered to be significant.

MLHFQ: Minnesota Living with Heart Failure Questionnaire; NYHA: New York Heart Association; SD: Standard deviation

The other parameters for which improvement was observed at 12 weeks included 6-min walk test, MLHFQ Score, NYHA class and peak VO_2_. The baseline mean distance covered in 6MWT in group 1 was 431 ± 64.79 and in group 2 was 418.8 ± 46.9 (p = 0.370), while at week 12, it was significantly more in group 1 (502.1 ± 70) as compared with group 2 (455.5 ± 52.3; p = 0.002). Similarly, the difference in MLHFQ score was not significant at baseline (p = 0.798) but significant at 12 weeks (p = 0.025). In the beginning, patients in the study were in a higher NYHA class (II, III) and at 12 weeks, none of them were in NYHA class III. This improvement was more marked in the FCM group than in the SOC group. Statistical analysis showed that at baseline, there was no difference in NYHA class (p = 0.232) but was significantly different at 12 weeks (p = 0.027), with benefit more marked in FCM group. In the same way, statistical analysis showed the difference in peak VO_2_ was not significant at baseline (p = 0.955) but significant at 12 weeks (p = 0.010). In contrast, ejection fraction (EF) did not show any significant improvement after 12 weeks. The mean EF in group 1 was 24.9% ± 5 and in group 2 was 25.8% ± 5.5 (p = 0.456). At 12 weeks, values in two groups were 26.6 ± 4.9 and 27.1 ± 5.4, respectively (p = 0.696). There was no significant difference in absolute VO_2_ at baseline (p = 0.730) and at 12 weeks (p = 0.079).

No serious side effects were noted during this study. There was no mortality or decompensated heart failure or severe hypersensitivity reaction to FCM. Minor side effects reported were constipation, abdominal discomfort, headache, metallic taste, myalgia and nausea, which were likely not due to FCM. There was no significant difference among two groups.

## Discussion

Recent studies have shown both clinical and statistical improvement with parenteral iron in patients with congestive heart failure. Limited data was available for the Indian population. Though patients included were younger than the other studies [4,7–9], there were no significant differences between the intervention groups. The prevalence of coronary artery disease observed was comparatively less, which may be attributable to our younger population included. Consistent with previously published evidence, our study showed significant improvement in serum ferritin values with FCM compared with placebo at the end of the time period (p < 0.0001). However, hemoglobin did not show any significant change with FCM. A similar finding was observed in FAIR-HF and FERRIC-HF, while EFFECT-HF showed improvement (p < 0.05). This nonsignificant increase may be because our study duration was short (12 weeks). We did not show any significant difference in EF at baseline (0.456) and at the end of the time period (p = 0.696). This observation was consistent with the other trials. However, walkable distance improved significantly in 6MWT at 12 weeks (p = 0.002). A similar finding was noted in CONFIRM-HF trial [8] where at 24 weeks they noted an increase in 6MWT distance by 18 + 8 m in FCM group, while decrease by 16 ± 8 in control group (p = 0.002). In FAIR-HF [7], consistent improvement starting from as early as 4 weeks and persisting at 12 and 24 weeks was seen (all p < 0.001). They noted mean treatment effect of 35 ± 8 m with FCM better than control.

At baseline, MLHFQ score was not different in two groups, 46.9 ± 15.7 in FCM group and 47.9 ± 15.1 in SOC group (p = 0.798). Okonko *et al.* [4] also used MLHFQ questionnaire in their study to assess quality of life in their study participants. At baseline, there was similar MLHFQ score in both groups (41 + 22 in iron group and 46 + 18 in control group). In CONFIRM-HF [8] and FAIR-HF [7], they used different parameters such as fatigue score, Kansas City Cardiomyopathy Questionnaire (KCCQ) score and EQ-5D Visual Analog Scale (EQ-5D VAS) score to assess quality of life. At the end of the time period, we found significant difference in MLHFQ score in both groups with FCM group noticing improved score than SOC group (34.7 ± 13 vs 41.9 ± 13.2, p = 0.025). Okonko *et al.* [4] also observed improvement in MLHFQ score in their treated group (31 + 25 vs 49 ± 28; p = 0.07). In CONFIRM-HF [8], patients in FCM group started experiencing improvement in fatigue score, KCCQ score, EQ-5D VAS score from 6th week which persisted through 52 weeks. In FAIR-HF [7], patients in FCM group started experiencing improvement in self-reported patient global assessment, EQ-5D VAS, KCCQ score from as early as 4th week, which persisted through 24th week (all p < 0.001). In summary, all above mentioned studies found significant improvement in quality of life who received parenteral iron supporting our conclusions.

In our study, when patients were reassessed at end of the study, they reported marked improvement in their functional class in FCM group than in SOC group (p = 0.027), which is in an agreement with other studies. In CONFIRM-HF [8], they noticed improvement in NYHA class in FCM group from 24th week onward persisting till end of study (p < 0.001). In EFFECT-HF [9], FCM group had improved their NYHA functional class significantly compared with patients in control group at 6, 12 and 24 weeks follow-up (all p < 0.05). Similarly, FAIR-HF [7] investigators found improvement in NYHA functional class in FCM group at week 24 (p < 0.001). In FERRIC-HF [4] also investigators reported improvement in NYHA class at 18th week in IV Iron group (p = 0.03), while in our study, patients in FCM group improved significantly in cardiopulmonary exercise testing (CPET) than in SOC group (p = 0.010). Our results are in congruence with finding in EFFECT-HF [9] study, but investigators in FERRIC-HF [4] differed with us. In order to remove the bias from weight, we measured absolute VO_2_ value (ml/min) which is independent of weight. We have not found any significant difference in two groups at baseline (p = 0.730) and at 12 weeks (p = 0.079). Okonko *et al.* [4] also found similar result in their study. Intravenous iron is a very safe molecule. In our study, we have not received any complaints of significant side effects. Other studies which have used injectable FCM such as CONFIRM-HF [8], FAIR-HF [7], EFFECT-HF [9] and FERRIC-HF [4] also did not report any significant side effect with FCM.

Apart from FCM, other formulations such as iron sucrose, low molecular weight dextran and iron isomaltoside are also available and have been studied in patients with heart failure. Iron sucrose [10] and low molecular weight iron dextran [11] have been shown to improve heart failure symptoms and improve echocardiographic parameters in patients with heart failure.

Iron isomaltoside 1000 [12] has provided symptomatic benefit in patients with heart failure in one study, while ferumoxytol [13] has proved its safety and efficacy in treating iron deficiency in patients with chronic kidney disease (CKD).

## Conclusion

Injectable iron causes marked improvement in functional status and quality of life of patients with chronic heart failure having iron deficiency. This improvement is evident from improved NYHA class, 6MWT distance covered and reduced MLHFQ score. Objective evidence of functional status like peak VO_2_ also improved with injectable iron. No improvement in EF was observed.

## Future perspective

Although our study showed improvement in symptoms and functional status, it failed to demonstrate any improvement in ejection fraction on echocardiogram. As poor left ventricular function is directly related to increased mortality, it is actually a setback to our belief that injectable iron may reduce long-term mortality in this population and in fact this is the biggest unanswered question of this treatment. There are also several other unresolved issues, which include effect on the patient of preserved ejection fraction heart failure, long-term implications of iron therapy and the need for better methods to select patients who can have maximal benefit rather than relying on ferritin and transferrin saturation. In the next decade, we need more studies to look for long-term outcomes and also look for downsides of using intravenous iron in heart failure population. One of these is ongoing, the ‘FAIR-HFpEF’ study. Hopefully this will throw some more light on this subject.
